# Characteristics of invasive *Acinetobacter* species isolates recovered in a pediatric academic center

**DOI:** 10.1186/s12879-016-1678-9

**Published:** 2016-07-22

**Authors:** Avish L. Jain, Christian M. Harding, Kaivon Assani, Chandra L. Shrestha, Mercedees Haga, Amy Leber, Robert S. Munson, Benjamin T. Kopp

**Affiliations:** Center for Microbial Pathogenesis, The Research Institute at Nationwide Children’s Hospital, Columbus, OH USA; Biomedical Sciences Graduate Program, College of Medicine, The Ohio State University, Columbus, OH USA; Department of Laboratory Medicine, Nationwide Children’s Hospital, Columbus, OH USA; Nationwide Children’s Hospital, Section of Pulmonary Medicine, 700 Children’s Drive, Columbus, OH 43205 USA

**Keywords:** Antibiotic resistance, Bacteremia, Pediatric, *Acinetobacter*

## Abstract

**Background:**

*Acinetobacter* species are associated with increasing mortality due to emerging drug-resistance. Pediatric *Acinetobacter* infections are largely undefined in developed countries and clinical laboratory identification methods do not reliably differentiate between members of the *Acinetobacter calcoaceticus-baumannii* complex, leading to improper identification. Therefore we aimed to determine risk factors for invasive *Acinetobacter* infections within an academic, pediatric setting as well as defining microbiologic characteristics of predominant strains.

**Methods:**

Twenty-four invasive *Acinetobacter* isolates were collected from 2009–2013. Comparative sequence analysis of the *rpoB* gene was performed coupled with phenotypic characterization of antibiotic resistance, motility, biofilm production and clinical correlation.

**Results:**

Affected patients had a median age of 3.5 years, and 71 % had a central catheter infection source. *rpoB* gene sequencing revealed a predominance of *A. pittii* (45.8 %) and *A. baumannii* (33.3 %) strains. There was increasing incidence of *A. pittii* over the study. Two fatalities occurred in the *A. pittii* group. Seventeen percent of isolates were multi-drug resistant. *A pittii* and *A. baumannii* strains were similar in motility, but *A pittii* strains had significantly more biofilm production (*P* value = 0.018).

**Conclusions:**

*A. pittii* was the most isolated species highlighting the need for proper species identification. The isolated strains had limited acute mortality in children, but the occurrence of more multi-drug resistant strains in the future is a distinct possibility, justifying continued research and accurate species identification.

**Electronic supplementary material:**

The online version of this article (doi:10.1186/s12879-016-1678-9) contains supplementary material, which is available to authorized users.

## Background

*Acinetobacter* species are Gram-negative coccobacilli many of which are found in soil and fresh water throughout our natural habitats [1]. However, certain *Acinetobacter* species are frequently isolated increasingly from healthcare facilities and are concomitantly the source of many nosocomial infections [1]. Specifically, *A. baumannii*, *A. pittii*, and *A. nosocomialis* of the *Acinetobacter calcoaceticus-baumannii* (*Acb*) complex have become the most medically relevant members of the genus as they are most frequently isolated from health care facilities as well as human tissues. Patients with impaired host defenses in intensive care unit (ICU) settings appear to be an at risk group of acquiring *Acinetobacter* infections [2]. Despite this knowledge, the infection source in outbreaks often cannot be determined, leaving recommendations to prevent future outbreaks limited [3]. Additionally, in recent years, some *Acinetobacter* strains have demonstrated a propensity to acquire resistance to multiple classes of antibiotics, rendering treatment of such hospitalized patients extremely difficult [4]. Furthermore, in pediatric patients, *A. baumannii* is thought to be the most prevalent organism, which is associated with bacteremia, ventilator-associated pneumonia, bronchopulmonary dysplasia, meningitis, and neutropenia [3, 5, 6].

Currently, our knowledge of the clinical impact of *Acinetobacter* infections in the pediatric patient population in developing countries and countries with suboptimal infection control resources have been well documented [2, 3, 7–11]. However, invasive *Acinetobacter* infections associated with large pediatric, academic institutes in developed countries are still poorly defined due to unclear risk factors. Recent studies documented risk factors for invasive infections as catheter insertions, prolonged use of antibiotics, as well as underlying chronic diseases [5, 12]. It is not clear if these risk factors are universal in all hospital settings, including locations outside of intensive care units. Additionally, improvements in bacterial sequencing, in particular the *Acinetobacter rpoB* gene*,* have allowed us to more accurately identify clinically important *Acinetobacter* species [13–15].

Therefore, the purpose of this study was to retrospectively review cases of invasive *Acinetobacter* infections occurring within an academic, pediatric setting in a developed country from a clinical perspective to define additional risk factors. Furthermore *Acinetobacter* strains were sequenced typed and preliminary characterized for motility and biofilm formation/maintenance. We hypothesized that *Acinetobacter* infections would be restricted to ICU settings and predominantly comprised of strains of *A. baumannii*.

## Methods

### Study design

The study was an observational review of patients with invasive *Acinetobacter* infections correlated with comparative sequencing of hyper-variable regions of the *rpoB* gene of *Acinetobacter* isolates. It was reviewed and approved by the Institutional Review Board of Nationwide Children’s Hospital (IRB14-00145). As aggregate patient data was used, individual consent was not obtained. The review population consisted of pediatric patients at a single, large, academic, pediatric institution identified during the years of 2009–2013. Inclusion criteria included pediatric patients less than 21 years old with at least one positive blood, bone, endotracheal, peritoneal, or cerebrospinal fluid culture result positive for *Acinetobacter* species [all identified as *A. baumannii* or *A. baumannii* complex by the clinical laboratory using Vitek 2 (bioMerieux, Durham NC) and other phenotypic methods as needed]*.* Antimicrobial susceptibility testing was performed on all isolates via the Vitek 2 using established breakpoints [16]. Multi-drug resistant isolates (MDR) was defined as isolates non-susceptible to ≥ 1 antimicrobial agent in ≥ 3 antimicrobial categories as defined by the joint initiative of the European Centre for Disease Prevention and Control (ECDC) and the Centers for Disease Control and Prevention (CDC) [17]. Study samples represented a convenience sample of existing isolates available in the clinical laboratory*.* Patients with cystic fibrosis respiratory isolates of *Acinetobacter* were excluded from the study. Isolates were frozen and stored at −80 °C.

A comprehensive review of clinical and demographic patient information was performed surrounding positive culture identification. Patient demographics included gender, age, race, county of residence, and insurance type. Information related to determining the source of the infection including the patient’s location within the hospital, source of the culture, and susceptibilities were recorded. A 48 h time period after cultures were obtained was chosen as an initial analysis point based on standard hospital time-based culture practices needed for determining culture results. At this time point antibiotic selection by the practitioner might change based on available antimicrobial susceptibilities. Underlying diagnoses, co-morbidities, secondhand smoke exposure, and vital signs were recorded. Laboratory data included complete blood count with differential, creatinine, liver function tests, as well as the next 5 culture results for each patient. C-reactive protein and erythrocyte sedimentation rates were available on less than 20 % of the cohort and therefore not included. Information regarding the patient’s hospital stay included length of stay, febrile status, oxygen use, ventilator use, imaging and invasive procedures, and mortality. Antibiotic treatment including antibiotics given and length taken by the patient along with antibiotic usage in the past 30 days were noted. Home medication including the use of chronic immunosuppressants, antibiotics, and gastric acid suppressants were all recorded.

### Comparative sequence analysis of the rpoB gene from clinical *Acinetobacter* isolates

*Acinetobacter rpoB* hyper-variable region sequencing was performed on *Acinetobacter* isolates according to a previously established protocol [18] with the following modifications. Genomic DNA (gDNA) was prepared from *Acinetobacter* isolates utilizing the Gentra Puregene Yeast/Bact. Kit B (Qiagen) according to the manufacturer’s protocol. One hundred nanograms of gDNA from each isolate were used in a PCR with one of two sets of primers. The first set of primers, spanning zone 1, were Ac696F (5′-TAYCGYAAAGAYTTGAAAGAAG-3′) and Ac1093R (5′-CMACACCYTTGTTMCCRTGA-3′) and the second set of primers, spanning zone 2, were Ac1055F (5′-GTGATAARATGGCBGGTCGT-3′) and Ac1598R (5′-CGBGCRTGCATYTTGTCRT-3′).

DreamTaq DNA polymerase (Thermo Scientific) was used according to manufacturer’s protocol with the following thermocycling conditions: 1 cycle at 95 °C for 2 min; 30 cycles at 95 °C for 30 s, 55 °C for 30 s, and 72 °C for 1 min; 1 cycle at 72 °C for 10 min. PCR products were purified using the QiaQuick PCR purification kit (Qiagen) according to the manufacturer’s protocol. Clean PCR products were verified by electrophoresis and sent off for sequencing by Eurofins MWG Operon. Raw sequence files were trimmed and edited using MegAlign and EditSeq software applications (DNASTAR). Trimmed sequence files for the two hyper-variable regions of a single isolate were combined into a single FASTA file for phylogenetic analysis. The combined FASTA files for each isolate, as well the corresponding sequences from the reference *Acinetobacter* strains used for phylogenetic analysis, were aligned initially by the Clustal W method utilizing UPGMB for the cluster method and the Kmer4-6 method for the distance measure. Later parameters utilized UPGMB for the cluster method and the Kimora % identity method for distance measure. Sequence distances were computed with the metric uncorrected pairwise distance and assembled into a phylogenetic tree using the MegAlign Pro software (DNASTAR).

### Motility assays

Twitching motility was investigated per previously described protocols with the following modifications [19]. Twitching plates contained 10 g tryptone/L and 10 g agarose/L. Briefly, bacterial strains were grown overnight and a single colony was used to inoculate each twitching plate to the bottom of the petri dish with a sterile wooden applicator stick. Twitching plates were incubated at 37 °C in a humidified incubator for 16 h. To visualize zones of twitching motility, the agarose was removed, the adherent bacterial population was washed with phosphate buffered saline, and stained with 0.1 % crystal in water. Bacteria positively exhibiting twitching motility were defined by demonstrating a zone of motility of >10 mm around the site of inoculation. Surface-associated motility was simultaneously assessed per our previously published methodologies [19]. Bacteria positively exhibiting surface-associated motility were defined as strains with a halo growth zone of >20 mm. Assays were performed in triplicate.

### Crystal violet retention assay

In order to assess the ability of each isolate to form biofilms, the crystal violet retention assay was performed as described previously [20] with minor modifications. Mueller Hinton (MH) broth was inoculated with one bacterial colony and incubated overnight at 37 °C for approximately 16–18 h. Cultures were subsequently diluted 1:100 in fresh MH broth and 100 μl of diluted culture was added to each well in 96 well microtiter plate and incubated overnight at 37 °C. Adherent cells were washed once with deionized water and stained with 125 μl of 0.1 % crystal violet solution for 15 min at room temperature and washed 5 times with deionized water. Subsequently, dye was released from the cells using 200 μl of 95 % ethanol. Absorbance was measured at 595 nm on a Synergy H1 Hybrid Reader spectrophotometer (Biotech, Biotech Instruments, Vermont, USA). The biofilm data represent the average of three independent experiments of triplicate wells.

### Statistical analysis

All analyses were performed using Stata/MP, version 13.1 or GraphPad Prism version 6.03. For all analyses, a *P* value < 0.05 was considered statistically significant. Descriptive statistics for continuous variables were presented as medians with 25–75^th^ percentile ranges; and descriptive statistics for categorical variables were presented as proportions. Mann-whitney tests were performed for sum comparisons.

## Results

### Bacterial isolates

**Fig. 1:**
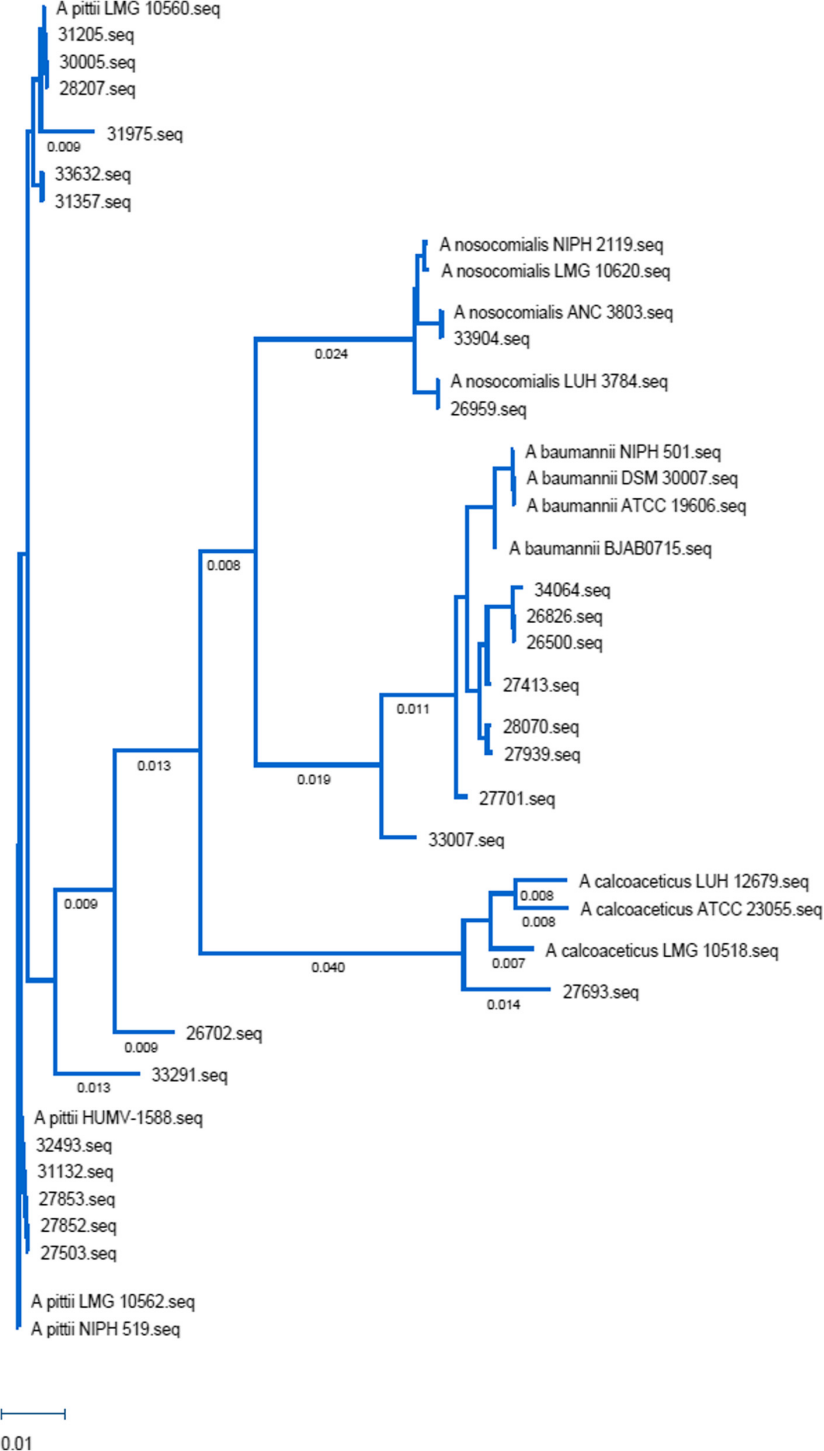
Phylogenetic Tree of Acinetobacter Clinical Isolates. *Acinetobacter rpoB* sequences from our collection as well as reference sequences were aligned using Clustal Omega based on the BioNJ algorithm. The tree was built using MegAlign Pro from the DNASTAR suite of programs. Horizontal branch length is a measure of genetic distance

Twenty-four isolates reported as *A. baumannii* from the clinical microbiology laboratory were recovered during the study period from 22 separate patients. A phylogenetic tree was derived after comparative sequence analysis of hyper-variable regions of the *rpoB* gene from *Acinetobacter* isolates using the BioNJ algorithm [21] (Fig. 1). The isolates were predominately composed of *A. pittii* (45.8 %) and *A. baumannii* (33.3 %). Other identified strains included one strain of *A. calcoaceticus* and two strains of *A. nosocomialis*. Two isolates, 26702 and 33291 were non-identified species. Both cases of *Acinetobacter*-associated death were caused by *A. pittii*. Notably, 70.0 % (*n* = 7) of the isolates in the last 2 years of the study were also *A. pittii,* representing a predominant shift towards *A. pittii* for the most recent institutional isolates (30.8 % *A. pittii* the previous three years combined, *n* = 4)*.* There were no reported hospital or unit specific outbreaks of *Acinetobacter* infection during the study period.

### Subject characteristics

Patient demographics and characteristics are listed in Table 1. The population was predominantly Caucasian and had a median age of 3.5 years. Identified risk factors for systemic infection are listed in Table 1. There was no consistent underlying primary diagnosis for the patients, as a total of 17 unique primary conditions were found for the 22 patients. Nearly 71 % of subjects had an indwelling central venous catheter source of the first positive culture with a non-significant increase in subjects with *A. pittii* (72.7 %) compared to *A. baumannii* (62.5 %). Over 40 % of subjects were on chronic antibiotics due to an immunosuppressive state at the time of *Acinetobacter* culture positivity, with nearly equal use between *A. baumannii* (50 %) and *A. pittii* (45.8 %) species. Complete data was available for all patients.

**Table 1:** Patient demographics and identified risk factors for *Acinetobacter* infection

	Percentage / Median	Range (if median reported)
Patient Demographics		
Male	62.5 %	
Caucasian	79.2 %	
Medicaid	70.8 %	
Age (years)	3.5	1.0–13.8
Body Mass Index (BMI)	18.0	15.8–21.2
Source		
Blood	79.2 %	
Peritoneal Fluid	4.2 %	
Bone/soft tissue	8.3 %	
Respiratory tract	8.3 %	
Risk Factors		
Previously healthy	0 %	
Central venous catheter	70.8 %	
Past bone marrow transplant	20.8 %	
Secondhand smoke exposure	29.2 %	
Taken antibiotics in past 30 days	41.7 %	
Chronic antibiotics >30 days	41.7 %	
Chronic immunosuppressants	37.5 %	
Gastroesophageal reflux	41.7 %	

### Hospital characteristics

Laboratory data and other characteristics relevant to the patients’ stay in the hospital are listed in Table 2. Markers of systemic kidney (creatinine) or liver (ALT, AST) dysfunction were not present for the majority of patients. Median length of stay for subjects was 10.5 days, with a non-significant increase in length of stay for *A. pittii* compared to *A. baumannii* (13.5 vs 3.5 days, *P* value = 0.13). Only two patients died during hospitalization, both with *A. pittii* infection. There was a significant increase in oxygen use for patients with *A. pittii* isolates compared to *A. baumannii* (54.5 % vs 0 %, *P* value = 0.018). Two-thirds of positive cultures were obtained in settings outside of the ICU, however 45.5 % of *A. pittii* isolates were from an ICU setting compared to 25.0 % *A. baumannii*. Median duration of antibiotic use was 14.0 days, including outpatient completion if subject discharged from hospital. Almost 80 % of repeat cultures were negative for *Acinetobacter* species within 48 h of initiating antibiotics. Antibiotic susceptibility patterns for the recovered isolates are shown in Table 3 as the percent of susceptible isolates to a given antibiotic. 72.7 % of A. *pittii* isolates were pan-susceptible, compared to 62.5 % of *A. baumannii* isolates (*P* value = 0.99). Additionally, the clinical laboratory designated 17 % (*n* = 4) of the isolates as MDR at the time of initial isolation, with three of these four strains from the *A. baumannii* group. Tobramycin demonstrated the least overall resistance (91.7 % strains susceptible, Table 3). Antibiotic selection for the first 48 h after clinical onset of infection and the final treatment course are shown in Table 4. Gentamicin was the most commonly used antibiotic for the full treatment course, although a wide variety of antibiotics were used during the first 48 h after onset of positive cultures (Additional file 1: Table S1).

**Table 2:** Patient laboratory parameters and hospital characteristics

	All isolates Percent/Median (Range)	*A. baumannii*	*A. pittii*
Laboratory Data			
White blood cell (k/mm^3^)	7.7 (1.6–14.3)	7.7 (1.2–11.0)	6.8 (1.5–20.9)
Hemoglobin (g/dL)	9.2 (7.8–9.9)	9.0 (7.6–9.3)	8.8 (7.8–11.1)
Neutrophils	60.0 % (44.0–76.0)	62.0 % (26.8–76.3)	57.0 % (49.5–69.5)
Creatinine (mg/dL)	0.45 (0.32–0.65)	0.44 (0.37–0.65)	0.35 (0.24–0.65)
ALT (IU/L)	37.5 (18.0–143.3)	13.0 (8.0–18.0)	135.0 (37.5–157.5)
AST (IU/L)	45.5 (38.5–93.5)	39.0 (37.0–41.0)	71.0 (45.0–177.0)
Hospital Visit			
Length of stay (days) Location of positive culture	10.5 (3.3–67.5)	3.5 (1.5–16.3)	13.5 (5.0–82.5)
Intensive care unit (ICU)	33.3 %	25.0 %	45.5 %
Hematology / Oncology unit	33.3 %	37.5 %	36.4 %
Emergency / Outpatient	33.3 %	37.5 %	18.1 %
Oxygen use	33.3 %	0 %	54.5 %
Ventilator use	37.5 %	18.2 %	45.5 %
Mortality	8.3 %	0 %	18.2 %
Antibiotic duration (days)	14.0 (9.3–14.0)	14.0 (10.0–19.0)	14.0 (6.0–14.0)
Follow up culture negative^a^	79.9 %	87.5 %	81.8 %

^a^Results of the next bacterial culture within 48 h of antibiotic initiation

**Table 3:** Antibiotic susceptibility patterns as percentage of susceptible *Acinetobacter* isolates

	All isolates (%, n)	*A. pittii* (%, n)	*A. baumannii* (%, n)
Ceftazidime	79.2, 19	90.1, 10	62.5, 5
Ciprofloxacin	87.5, 21	100, 11	62.5, 5
Gentamicin	83.3, 20	90.1, 10	62.5, 5
Meropenem	87.5, 21	100, 11	62.5, 5
Piperacillin-Tazobactam	87.5, 21	100, 11	62.5, 5
Trimethoprim-sulfamethoxazole	83.3, 20	90.1, 10	75.0, 6
Tobramycin	91.7, 22	100, 11	75.0, 6

**Table 4:** Phenotypic characteristics of *Acinetobacter* isolates association with clinical factors

	Biofilm-forming (*n* = 17)	Non-biofilm-forming (*n* = 7)	*P* value	Swarming motility (*n* = 16)	Non-swarming motility (*n* = 8)	*P* value	Twitching motility (*n* = 14)	Non-twitching motility (*n* = 10)	*P* value
Age (years)	4.0 (0.4–11.5)	3.0 (2.0–19.0)	0.84	4.5 (1.0–16.8)	3.5 (0.9–4.0)	0.37	3.5 (0.6–17.5)	3.5 (1.5–6.3)	0.57
Central line infection	64.7 %	85.7 %	0.38	68.8 %	66.7 %	0.99	71.4 %	70.0 %	0.99
Bacteremia at 48 h	17.6 %	0.0 %	0.53	12.5 %	12.5 %	0.99	14.3 %	10.0 %	0.99
MDR	5.9 %	42.9 %	0.027	18.8 %	12.5 %	0.80	21.4 %	10.0 %	0.48
ICU	35.3 %	28.6 %	0.76	37.5 %	25.0 %	0.56	42.9 %	20.0 %	0.26
Abx in past 30 days	47.1 %	28.6 %	0.43	37.5 %	50.0 %	0.67	42.9 %	40.0 %	0.99
Immuno-suppressant	29.4 %	71.4 %	0.06	43.8 %	37.5 %	0.78	42.9 %	40.0 %	0.99
Length of stay (days)	17.0 (4.5–80.0)	5.0 (3.0–20.0)	0.21 0.99	18.5 (4.3–63.3)	5.0 (3.3–35)	0.32	11.0 (3.0–97.5)	10.5 (4.5–27.3)	0.92
SHS exposure	23.5 %	28.6 %	0.99	34.1 %	53.5 %	0.13	28.6 %	20.0 %	0.99
GER	35.3 %	28.6 %		43.8 %	12.5 %	0.14	35.7 %	30.0 %	0.99

Data are expressed as a percentage of cases with medians and ranges

*Abx* antibiotic use in 30 days prior to infection; *GER* gastroesophageal reflux; *ICU* Intensive care unit care required; *MDR* multi-drug resistance with isolates non-susceptible to ≥ 1 antimicrobial agent in ≥ 3 antimicrobial categories; *SHS* secondhand smoke

### Acinetobacter isolate motility

**Fig. 2:**
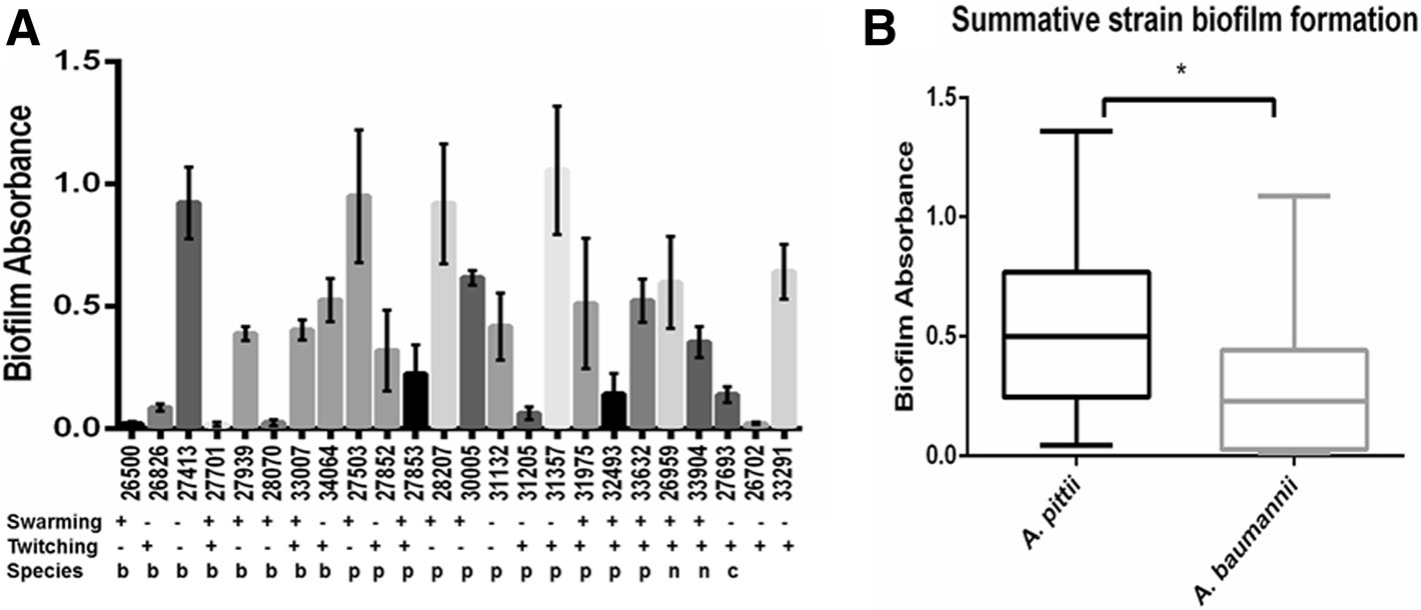
Bacterial isolate characteristics. **a** Individual isolate results of biofilm and twitching and surface-associated motility assays with grouping by species. The level of biofilm formation was determined by measuring the absorbance at 595 nm post crystal violet staining. Error bars represent the standard deviation of the mean of three independent experiments. Twitching and surface-associated motility are characterized as positive or negative based on experimental methods. All experiments performed in triplicate. Strains are designated as: “b” = *baumannii,* “p” = *pittii*, “n” = *nosocomialis*, “c” = *calcoaceticus,* two strains are undesignated. **b** Box and whiskers plot demonstrating sum of median biofilm production with minimum and maximum error bars for *A. pittii* and *A. baumannii* strains from Fig. 2a, *P* value =0.018

Twitching and surface-associated motility (swarming) have been shown to be distinct phenotypes for medically relevant *Acinetobacter* [19, 22], but have not been described for *A. pittii*. All isolated strains were assessed for motility characteristics (Fig. 2a). Surface-associated motility was observed in 63.6 % of the *A. pittii* strains, and 62.5 % of the *A. baumannii* strains (*P* value = 0.99). Twitching motility was observed in 72.7 % of the *A. pittii* strains, and 50 % of the *A. baumannii* strains (*P* value = 0.37). Both phenotypes were present in 45.5 % of the *A. pittii* strains, and only 25 % of the *A. baumannii,* with the four most recently isolated *A. pittii* strains harboring both phenotypes*.* The *A. calcoaceticus* strain demonstrated twitching, but not swarming motility.

### Biofilm formation

The production of biofilms allow for bacterial persistence and adherence to medical devices and vascular lines [23]. Because over 70 % of our isolates were associated with central line infections, we wanted to determine if there were any differences in the capability of the isolates to form biofilms, which may predispose to local strain emergence on medical devices. To quantitate *Acinetobacter* strain biofilm capabilities, individual strains were tested for their ability to adhere to microtiter plates as determined by crystal violet stain retention. *A. pittii* isolates had significantly higher median biofilm production compared to *A. baumannii* isolates (Fig. 2b, *P* value = 0.0071*.* Furthermore, both *A. nosocomialis* isolates (#26959, # 33904) demonstrated an intermediate ability to form biofilms as determined by crystal violet retention, however, the *A. calcoaceticus* isolate (#27693) demonstrated a minimal ability to form a biofilm (Fig. 2a)*.* Overall, 75 % of all isolates were able to form biofilms.

### Clinical characteristics based on phenotypic expression

After *Acinetobacter* isolate motility and biofilm formation studies were performed, isolates were than characterized for relevant clinical outcomes based on the percentage of isolates expressing each phenotype (Table 4). There was no difference in biofilm production between central line-associated and non-central line-associated isolates (median absorbance 0.35 vs 0.42, *P* value = 0.62), although there was a non-statistically significant increase in non-biofilm producing isolates associated with central line infections (85.7 vs 64.7 %, *P* value = 0.38). However, all isolates that demonstrated persistent bacteremia at 48 h were biofilm-producers. Multi-drug resistance was present in both biofilm and non-biofilm producing isolates, but MDR isolates were predominantly non-biofilm producers (42.9 vs 5.9 %, *P* value = 0.027). Chronic immunosuppressant use was greater in non-biofilm producing isolates, but this did not reach statistical significance (71.4 vs 29.4 %, *P* value = 0.06). There was no difference in the requirement for ICU care, secondhand smoke exposure, or use of proton pump inhibitors for gastroesophageal reflux based on biofilm formation. There were no discernible differences between twitching and swarming isolates in any of the clinical characteristics.

## Discussion

Members of the *Acinetobacter calcoaceticus-baumannii* complex are regarded as opportunistic human pathogens of increasing relevance worldwide due in part to the emergence of multiply-drug resistant strains; however, the description of pathogenic *Acb* members other than *A. baumannii* remains limited due in part technological limitations with current clinical laboratory identification methods [24]*.* Although emerging methods such as MALDI-ToF analysis may aid in clinical laboratory-based identification *Acb* members for the future, these methods have not become the gold-standard even in developed countries. In this study we report the emergence of *A. pittii* strains from pediatric patients with invasive infections, along with a relatively low mortality rate.

Outbreaks of *A. pittii* have only been previously reported to a low extent and were thought to be rare in pediatric settings in the United States [5, 8, 9, 25]. This may be attributed to the fact that most of the common clinical laboratory identification methods do not reliably differentiate between members of the *Acb*; hence, many publications may in fact be referring to all members of the *Acb* when specifically referencing *A. baumannii* [5, 24]. Importantly, in the documented cases where *A. pittii* has been reported, it can be more commonly recovered from samples than *A. baumannii* [8], but this is not the case in all settings. In our isolate collection, there was an emergence of *A. pittii* over the last two years of the study period, indicating a shift towards *A. pittii* over *A. baumannii.* This finding will require further follow-up due to the short time-frame of study follow-up. The significant rise in the number of isolates of *A. pittii* in a pediatric population also signifies a potential new trend to be cognizant of for clinicians and researchers, due largely to differences in antibiotic resistance profiles between species. Notably, we did not see a difference in basic microbiologic characteristics such as surface motility between the *A. pittii* and *A. baumannii* strains. However, the majority of the most recent *A. pittii* strains did display both twitching and surface-associated motility phenotypes, indicating the likely expression of a functional type IV pilus as well as changing local population dynamics. Additionally*, A pittii* isolates averaged more robust biofilm production when compared to *A. baumannii* isolates, indicating another potential pathogenic mechanism for the increasing *A pittii* prevalence. However, laboratory biofilm studies may not reflect human in vivo biofilm formation, therefore further studies are warranted in this area for the future.

Despite previous reports of high mortality associated with antibiotic resistant *Acinetobacter* strains [5, 7], in our sampling only 2 out of 24 cases were associated with patient deaths. Notably, both deaths were from *A. pittii*, again in contrast with the existing pediatric literature on *A. baumannii*-associated mortality, and the antibiotic resistance patterns observed. We postulate that one difference in the overall low mortality rates could be the relative sensitivity to the aminoglycoside antibiotics coupled with prompt administration, as this class of antibiotic was utilized in the first 48 h of a suspected infection in half of the reported cases. There was also a low prevalence of MDR strains. Both of these factors may have contributed to the fact that nearly 80 % of the strains were cleared from the systemic circulation within 48 h of initiating antibiotics. Persistent bacteremia was noted to be associated with biofilm-producing isolates, which is of importance when assessing therapeutic options for persistent *Acinetobacter* infections. Additionally, compared to other reported pediatric *Acinetobacter* infections, the isolated strains demonstrated lower overall morbidity as reflected by fewer intensive care requirements and less multiple organ involvement. Much of the published pediatric literature focuses on neonatal intensive care units with multiple *Acinetobacter* infections [3, 8–12]. However, in our sample, the median age was 3.5 years with no reported neonatal infections. We were unable to assess for the role of existing institutional infection control bundles in preventing ICU-associated infections during this retrospective study. Therefore, continued work will be needed to address potential epidemiologic factors associated with pediatric *Acinetobacter* infections in the United States.

Other species of *Acinetobacter* are increasingly recognized to cause disease in humans such as *A. bereziniae* [26], further supporting the assumption that improved species identification of *Acinetobacter* infections may be needed to accurately follow emerging infectious trends from an epidemiologic level. These results further highlight the medical relevance of the *Acinetobacter* genus as whole, where an encounter between a patient with a compromised immune system and a species of *Acinetobacter*, regardless of its characterized pathogenicity can result in infection.

We acknowledge that this study has several limitations. First, the study was retrospective in nature, therefore limiting the availability of some clinical information. Second, the small sample size, 5-year time frame, and single-center setting limits the generalizability of the findings and analysis of isolate phenotypic characteristics in relation to clinical outcomes. However, the samples may be more representative of U.S. pediatric academic centers than previous reports from developing countries. Finally, the clinical laboratory did not report antibiotic susceptibilities for all classes of antibiotics for the four isolates obtained in the first year of the study.

## Conclusions

In summary, knowledge of pediatric *Acinetobacter* infections has been limited to date by clinical laboratory identification methods, which may presume *Acinetobacter* induced infections to be caused by *A. baumannii. A. pittii* was discovered to be the dominant local clinical strain expressing a variety of phenotypic characteristics including 2 fatalities. The isolated strains in this study had limited overall long-term morbidity or mortality in a pediatric population, but the occurrence of more MDR strains in the future is a distinct possibility, leading to the need for continued research and accurate species identification.

## Abbreviations

*Acb, Acinetobacter calcoaceticus-baumannii;* gDNA, genomic DNA; ICU, intensive care unit; MH, Mueller Hinton

## Additional files

Additional file 1: Table S1. Frequency of antibiotic regimens utilized during treatment of infected patients. Description: Breakdown of antibiotic choices utilized clinically during the first 48 h of suspected Acinetobacter infection as well as the final treatment choices. (DOC 33 kb) Additional file 2:
*rpoB* sequences from *Acinetobacter* isolates. Description: Full *rpoB* sequences from each *Acinetobacter* isolate in the study. (DOC 41 kb)
