# Supplementary material for: Characteristics of invasive Acinetobacter species isolates recovered in a pediatric academic center
Source: BMC Infect Dis. 2016 Jul 22;16:346. doi: 10.1186/s12879-016-1678-9 (PMC4957376; doi:10.1186/s12879-016-1678-9)
Supplement: Additional file 1: Table S1. — Frequency of antibiotic regimens utilized during treatment of infected patients. Description: Breakdown of antibiotic choices utilized clinically during the first 48 h of suspected Acinetobacter infection as well as the final treatment choices. (DOC 33 kb) [file 12879_2016_1678_MOESM1_ESM.doc]

**Table S1 Frequency of antibiotic regimens utilized during treatment of infected patients**

| **Antibiotics*** | **Initial 48 hours** | **Full Treatment Course** |
| --- | --- | --- |
| Gentamicin | 37.50% | 45.80% |
| Meropenem | 16.70% | 16.70% |
| Ampicillin/Sulbactam | 4.20% | 16.70% |
| Piperacillin/Tazobactam | 41.70% | 12.50% |
| Ceftazidime | 12.50% | 8.30% |
| Ciprofloxacin | 12.50% | 8.30% |
| Minocycline | 4.20% | 4.20% |
| Azithromycin | 4.20% | 4.20% |
| Cefepime | 0% | 4.20% |
| Colistin | 0% | 4.20% |
| Amikacin | 12.50% | 0% |
| Ceftriaxone | 8.30% | 0% |
| Levofloxacin | 4.20% | 0% |

*Antibiotic usage during the first 48 hours of suspected infection (after culture obtained) and for full treatment course are reported. Antibiotics are grouped in descending order of frequency of use for full treatment course.
