# Supplementary material for: Characteristics of invasive Acinetobacter species isolates recovered in a pediatric academic center
Source: BMC Infect Dis. 2016 Jul 22;16:346. doi: 10.1186/s12879-016-1678-9 (PMC4957376; doi:10.1186/s12879-016-1678-9)
Supplement: Additional file 2: — rpoB sequences from Acinetobacter isolates. Description: Full rpoB sequences from each Acinetobacter isolate in the study. (DOC 41 kb) [file 12879_2016_1678_MOESM2_ESM.doc]

**Additional file 2 *rpoB* sequences from *Acinetobacter* isolates**

*>Acinetobacter* 26500

CGGCTCGTGAGCGTGTAATTCGTTTGCTTAAAGGCCAAGAGTCTAATGGCGGTGGTTCAACTAAACGTGGTGATAAACTTTCTGAAGATTTATTATCTGGTTTAGAGCTTGTTGACTTACTTGAAATTCAACCAGCAGATGAAGCGATCGCTGAGCGTTTAACTCAAATTCAAGTGTTCTTGAAAGAGAAGAGCGCAGAAATCGATGAGAAATTCGCTGAGAAGAAACGTAAGCTTGCAACAGGTGATGAATTAACAACTGGCGTATTAAAAGTTGTTAAAGTTTACTTAGCTGTTAAACGTCGTATTCATCTTACCTGTTGAAGACATGCCACATGATGCTAACGGTGTGCCGGTAGATATCGTATTGAACCCGCTAGGTGTACCATCTCGTATGAACGTGGGTCAGATTCTAGAGACTCACTTGGGTATGGCGGCTAAAGGGCTTGGTGACAAAATCGAAAAAATGTTGAAAGAACAACGTACAGTTTTAGAACTGCGCGAATTCTTAGACAAGATTTATAACAAAGTCGGCGGTGAGCAAGAAGATCTTGATAGCTTGACTGATGAAGAAATTCTAGCGCTTGCAGGTAACTTGCGTGCGGGTGTGCCTTTAGCTACTCCGTATTTGATGGTGCTGAAGAAAGTCAAATTAAAGACTTACTTGAATTGGCTGACATTTCACGTACGGGTCAAACAGTATTGTTTGACGGACGTACAGGTGAACAGTTTGACCGTCCAGTAACTGT

>*Acinetobacter* 26702

CTCGTGAGCGTGTAATTCGTTTGCTTAAAGGCCAAGAGTCTAATGGCGGTGGTTCAACTAAACGTGGTGACAAACTTGTTGAAGAAGTGTTATCTGGTTTAGAGCTTGTTGATTTACTTGAAATTCAACCCGCAGATGAAGCAATCGCTGAGCGTTTAACTCAAATTCAAGTGTTCTTAAAAGAAAAGAGCGCAGAAATTGATGAGAAATTCGCTGAGAAGAAACGTAAGCTTGCAACAGGTGATGAATTAACAACTGGCGTATTGAAAGTTGTTAAAGTTTACTTAGCTGTTAAACGTCGTATTCATCTAACATCTTACCTGTTGAAGACATGCCACACGATGCTAACGGTGTGCCGGTAGATATCGTATTGAACCCGTTGGGCGTACCATCTCGTATGAACGTGGGTCAGATTCTTGAGACTCACTTAGGTATGGCGGCTAAAGGGCTTGGTGATAAAATCGAGAAAATGTTGAAAGAACAACGTACAGTTTTAGAACTGCGTGAATTCTTAGACAAGATTTATAACAAAGTCGGTGGTGAGCAAGAAGATCTTGATAGCTTAACTGATGATGAAATCTTAGCACTTTCAGGCAACTTGCGTGCCGGTGTACCTTTGGCTACTCCTGTATTCGATGGTGCTGAAGAAAGTCAAATTAAAGACTTACTTGAGTTAGCTGGTATTTCACGTACAGGTCAAACAGTATTGTATGATGGCCGTACAGGTGAACAGTTTGATCGTCCTGTAACTGTAGG

>*Acinetobacter* 26826

GCGGCTCGTGAGCGTGTAATTCGTTTGCTTAAAGGCCAAGAGTCTAATGGCGGTGGTTCAACTAAACGTGGTGATAAACTTTCTGAAGATTTATTATCTGGTTTAGAGCTTGTTGACTTACTTGAAATTCAACCAGCAGATGAAGCGATCGCTGAGCGTTTAACTCAAATTCAAGTGTTCTTGAAAGAGAAGAGCGCAGAAATCGATGAGAAATTCGCTGAGAAGAAACGTAAGCTTGCAACAGGTGATGAATTAACAACTGGCGTATTAAAAGTTGTTAAAGTTTACTTAGCTGTTAAACGTCGTATTCATCTTACCTGTTGAAGACATGCCACATGATGCTAACGGTGTGCCGGTAGATATCGTATTGAACCCGCTAGGTGTACCATCTCGTATGAACGTGGGTCAGATTCTAGAGACTCACTTGGGTATGGCGGCTAAAGGGCTTGGTGACAAAATCGAAAAAATGTTGAAAGAACAACGTACAGTTTTAGAACTGCGCGAATTCTTAGACAAGATTTATAACAAAGTCGGCGGTGAGCAAGAAGATCTTGATAGCTTGACTGATGAAGAAATTCTAGCGCTTGCAGGTAACTTGCGTGCGGGTGTGCCTTTAGCTACTCCTGTATTTGATGGTGCTGAAGAAAGTCAAATTAAAGACTTACTTGAATTGGCTGACATTTCACGTACGGGTCAAACAGTATTGTTTGACGGACGTACAGGTGAACAGTTTGACCGTCCAGTAACTGT

>*Acinetobacter*  26959

CGTGTAATCCGTTTACTTAAAGGCCAAGAGTCTAATGGCGGTGGTTCAACTAAACGTGGCGATAAACTTTCTGAAGATTTATTGTCTGGTTTAGAGCTTGTTGATTTACTTGAAATTCAACCGACAGATGAAGCAATCGCTGAGCGTTTAACTCAGATTCAAGTGTTCTTGAAAGAGAAGAGCGCAGAAATCGATGAGAAATTCGCTGAGAAGAAACGTAAGCTTGCAACAGGTGATGAGCTAACAACTGGCGTATTGAAAGTTGTTAAAGTTTACTTAGCTGTTAAACGTCGTATTCATCTTACCTGTTGAAGACATGCCACACGATGCGAACGGTGTACCTGTAGATATCGTATTGAACCCGTTGGGTGTACCATCTCGTATGAACGTGGGTCAGATTCTCGAGACTCACTTGGGTATGGCGGCTAAAGGGCTTGGTGACAAAATCGAAAAAATGTTGAAAGAACAGCGTACAGTTTTAGAACTACGTGAATTCTTAGACAAGATTTATAACAAAGTCGGTGGCGAGCAAGAAGATCTTGATAGCTTGACTGATGATGAAATCCTGGCACTTTCAGGCAACTTGCGTGCTGGTGTTCCTTTGGCTACTCCTGTATTTGATGGTGCTGAAGAAAGTCAAATTAAAGATTTACTTGAGTTGGCTGACATTTCACGTACAGGTCAAACAGTATTGTTTGATGGACGTACAGGTGAACAGTTTGACCGTCCAGTAACTGT

>*Acinetobacter* 27412

CGGCTCGTGAGCGTGTAATCCGTTTACTTAAAGGCCAAGAGTCTAATGGCGGTGGTTCAACTAAACGTGGTGATAAACTTTCTGAAGATTTATTATCTGGTTTAGAGCTTGTTGACTTACTTGAAATTCAACCAGCAGATGAAGCAATCGCTGAGCGTTTAACTCAAATTCAAGTGTTCTTGAAAGAGAAGAGCGCAGAAATCGATGAGAAATTCGCTGAGAAGAAACGTAAGCTTGCAACAGGTGATGAATTAACAACTGGCGTATTAAAAGTTGTTAAAGTTTACTTAGCTGTTAAACGTCGTATTCAGCTCTTACCTGTTGAAGACATGCCACATGATGCTAACGGTGTGCCGGTAGATATCGTATTGAACCCGCTGGGTGTACCATCTCGTATGAACGTGGGTCAGATTCTAGAGACTCACTTGGGTATGGCGGCTAAAGGGCTTGGTGACAAAATCGAAAAAATGTTGAAAGAACAACGTACAGTTTTAGAACTGCGCGAATTCTTAGACAAGATTTATAACAAAGTCGGCGGTGAGCAAGAAGATCTTGATAGCTTGACTGATGAAGAAATTCTAGCGCTTGCAGGTAACTTGCGTGCGGGTGTGCCTTTAGCTACTCCTGTATTTGATGGTGCTGAAGAAAGTCAAATTAAAGACTTACTTGAATTGGCTGACATTTCACGTACTGGTCAAACAGTATTGTTTGACGGACGTACAGGTGAACAGTTTGACCGTCCAGTAACTGT

>*Acinetobacter* 27503

CAAGAGTCTAATGGCGGTGGTTCAACTAAACGTGGTGACAAACTCGTTGAAGAAGTGTTATCTGGTTTAGAGCTTGTTGATTTACTTGAAATTCAACCGGCAGATGAAGCAATCGCTGAGCGTTTAACTCAAATTCAAGTGTTCTTAAAAGAAAAGAGCGCAGAAATTGACGAGAAATTCGCTGAGAAGAAACGTAAGCTTGCAACAGGTGATGAATTAACAACTGGCGTATTGAAAGTTGTTAAAGTTTACTTAGCTGTTAAACGTCGTCTTACCTGTTGAAGACATGCCACACGATGCTAACGGTGTACCAGTAGATATCGTATTGAACCCGTTGGGCGTACCATCTCGTATGAACGTGGGTCAGATTCTTGAGACTCACTTAGGTATGGCGGCTAAAGGGCTTGGTGATAAAATCGAGAAAATGTTGAAAGAACAGCGTACAGTTTTAGAACTGCGTGAATTCTTAGACAAGATTTATAACAAAGTCGGTGGTGAGCAAGAAGATCTTGATAGCTTAACTGATGCTGAAGTCTTGGCACTTTCAGGCAACTTACGTGCTGGTGTACCTTTGGCTACTCCTGTATTCGATGGTGCTGAAGAAAGTCAAATTAAAGACTTGCTTGAGTTAGCTGGTATCTCTCGTACAGGTCAAACAGTATTGTTTGATGGCCGTACTGGTGAACAGTTTGATCGTCCTGTAACTGTAGGTTAC

>*Acinetobacter* 27693

GCTCGTGAGCGTGTAATTCGTTTGCTTAAAGGCCAAGAATCTAACGGTGGTGGTTCGACTAAACGTGGCGACAAACTTGTTGAAGATATGTTGTCTGGTTTAGAGCTTGTTGACTTACTTGAAATCCAACCTACAGACGAAGCAATTGCTGAACGTTTATCTCAAATTCAAGTGTTCTTGAAAGAGAAGAGCGCAGAAATTGATGAGAAGTTTGCAGAGAAGAAACGTAAGCTTTCGACTGGTGATGAGTTAACAACAGGCGTTCTGAAAGTTGTTAAAGTTTACCTAGCAGTTAAACGTCGCATTCATTTTACCTGTTGAAGACATGCCACACGATGCGAACGGTGTACCAGTAGATATCGTATTGAACCCATTGGGTGTACCATCTCGTATGAACGTGGGTCAGATTCTTGAGACTCACTTGGGTATGGCGGCTAAAGGGCTTGGCGATAAAATCGAAAAAATGTTGAAAGAACAGCGTACAGTTTTAGAACTTCGCGAATTCTTAGACAAGATTTATAACAAAGTCGGTGGCGAGCAAGAAGATCTTGATAGCTTAACTGATGCTGAAGTTTTAGCTCTTTCTGGCAACTTACGTGCTGGTGTGCCTTTAGCTACTCCTGTATTTGATGGTGCTGAAGAAAGCCAAATTAAAGACTTACTTGAATTAGCTGACATTTCACGTACTGGTCAAACAGTATTGTTTGATGGTCGTACAGGTGAACAGTTTGATCGTCCTGTAACTGTAGG

>*Acinetobacter* 27701

CGGCTCGTGAGCGTGTAATTCGTTTGCTTAAAGGCCAAGAGTCTAATGGCGGTGGTTCAACTAAACGTGGTGATAAACTTTCTGAAGATTTATTATCTGGTTTAGAGCTTGTTGACTTACTTGAAATTCAACCAGCAGATGAAGCAATCGCTGAGCGTTTAACTCAAATTCAAGTGTTCTTAAAAGAGAAGAGCGCAGAAATCGATGAGAAATTCGCTGAGAAGAAACGTAAGCTTGCAACAGGTGATGAATTAACAACTGGCGTATTGAAAGTTGTTAAAGTTTACTTAGCTGTTAAACGTCGTATTCATCTTACCTGTTGAAGACATGCCACATGATGCTAACGGTGTGCCGGTAGATATCGTATTGAACCCGCTGGGTGTACCATCTCGTATGAACGTGGGTCAGATTCTAGAGACTCACTTGGGTATGGCGGCTAAAGGGCTTGGTGACAAAATCGAAAAAATGTTGAAAGAACAACGTACAGTTTTAGAACTGCGCGAATTCTTAGACAAGATTTATAACAAAGTCGGTGGTGAGCAAGAAGATCTTGATAGCTTGACTGATGAAGAAATTCTAGCGCTTGCAGGTAACTTGCGTGCGGGTGTGCCTTTAGCTACTCCTGTATTTGATGGTGCTGAAGAAAGTCAAATTAAAGACTTACTTGAATTGGCTGACATTTCACGTACTGGTCAAACAGTATTGTTTGACGGACGTACAGGTGAACAGTTTGACCGTCCAGTAACTGT

>*Acinetobacter* 27852

CTCGTGAGCGTGTAATTCGTTTGCTTAAAGGCCAAGAGTCTAATGGCGGTGGTTCAACTAAACGTGGTGACAAACTCGTTGAAGAAGTGTTATCTGGTTTAGAGCTTGTTGATTTACTTGAAATTCAACCGGCAGATGAAGCAATCGCTGAGCGTTTAACTCAAATTCAAGTGTTCTTAAAAGAAAAGAGCGCAGAAATTGACGAGAAATTCGCTGAGAAGAAACGTAAGCTTGCAACAGGTGATGAATTAACAACTGGCGTATTGAAAGTTGTTAAAGTTTACTTAGCTGTTAAACGTCGTATTCATTACCTGTTGAAGACATGCCACACGATGCTAACGGTGTACCAGTAGATATCGTATTGAACCCGTTGGGCGTACCATCTCGTATGAACGTGGGTCAGATTCTTGAGACTCACTTAGGTATGGCGGCTAAAGGGCTTGGTGATAAAATCGAGAAAATGTTGAAAGAACAGCGTACAGTTTTAGAACTGCGTGAATTCTTAGACAAGATTTATAACAAAGTCGGTGGTGAGCAAGAAGATCTTGATAGCTTAACTGATGCTGAAGTCTTGGCACTTTCAGGCAACTTACGTGCTGGTGTACCTTTGGCTACTCCTGTATTCGATGGTGCTGAAGAAAGTCAAATTAAAGACTTGCTTGAGTTAGCTGGTATCTCTCGTACAGGTCAAACAGTATTGTTTGATGGCCGTACTGGTGAACAGTTTGATCGTCCTGTAACTGTAGGTTACA

>*Acinetobacter* 27853

CTCGTGAGCGTGTAATTCGTTTGCTTAAAGGCCAAGAGTCTAATGGCGGTGGTTCAACTAAACGTGGTGACAAACTCGTTGAAGAAGTGTTATCTGGTTTAGAGCTTGTTGATTTACTTGAAATTCAACCGGCAGATGAAGCAATCGCTGAGCGTTTAACTCAAATTCAAGTGTTCTTAAAAGAAAAGAGCGCAGAAATTGACGAGAAATTCGCTGAGAAGAAACGTAAGCTTGCAACAGGTGATGAATTAACAACTGGCGTATTGAAAGTTGTTAAAGTTTACTTAGCTGTTAAACGTCGTATTCATCTAACATCTTACCTGTTGAAGACATGCCACACGATGCTAACGGTGTACCAGTAGATATCGTATTGAACCCGTTGGGCGTACCATCTCGTATGAACGTGGGTCAGATTCTTGAGACTCACTTAGGTATGGCGGCTAAAGGGCTTGGTGATAAAATCGAGAAAATGTTGAAAGAACAGCGTACAGTTTTAGAACTGCGTGAATTCTTAGACAAGATTTATAACAAAGTCGGTGGTGAGCAAGAAGATCTTGATAGCTTAACTGATGCTGAAGTCTTGGCACTTTCAGGCAACTTACGTGCTGGTGTACCTTTGGCTACTCCTGTATTCGATGGTGCTGAAGAAAGTCAAATTAAAGACTTGCTTGAGTTAGCTGGTATCTCTCGTACAGGTCAAACAGTATTGTTTGATGGCCGTACTGGTGAACAGTTTGATCGTCCTGTAACTGTAGGTTACA

>*Acinetobacter* 27939

AGCGGCTCGTGAGCGTGTAATTCGTTTGCTTAAAGGCCAAGAGTCTAATGGCGGTGGTTCAACTAAACGTGGTGATAAACTTTCTGAAGATTTATTATCTGGTTTAGAGCTTGTTGACTTACTTGAAATTCAACCAGCAGATGAAGCAATCGCTGAGCGTTTAACTCAAATTCAAGTGTTCTTGAAAGAGAAGAGCGCAGAAATCGATGAGAAATTCGCTGAGAAGAAACGTAAGCTTGCAACAGGTGATGAATTAACAACTGGTGTATTGAAAGTTGTTAAAGTTTACTTAGCTGTTAAACGTCGTTACCTGTTGAAGACATGCCACATGATGCTAACGGTGTGCCGGTAGATATCGTATTGAACCCGCTGGGTGTACCATCTCGTATGAACGTGGGTCAGATTCTAGAGACTCACTTGGGTATGGCGGCTAAAGGGCTTGGTGACAAAATCGAAAAAATGTTGAAAGAACAACGTACAGTTTTAGAACTGCGCGAATTCTTAGACAAGATTTATAACAAAGTCGGCGGTGAGCAAGAAGATCTTGATAGCTTGACTGATGAAGAAATTCTAGCGCTTGCAGGTAACTTGCGTGCGGGTGTGCCTTTAGCTACTCCTGTATTTGATGGTGCTGAAGAAAGTCAAATTAAAGACTTACTTGAATTGGCTGACATTTCACGTACGGGTCAAACAGTATTGTTTGACGGACGTACAGGTGAACAGTTTGACCGTCCAGTAACTGT

>*Acinetobacter* 28070

CGGCTCGTGAGCGTGTAATTCGTTTGCTTAAAGGCCAAGAGTCTAATGGCGGTGGTTCAACTAAACGTGGTGATAAACTTTCTGAAGATTTATTATCTGGTTTAGAGCTTGTTGACTTACTTGAAATTCAACCAGCAGATGAAGCAATCGCTGAGCGTTTAACTCAAATTCAAGTGTTCTTGAAAGAGAAGAGCGCAGAAATCGATGAGAAATTCGCTGAGAAGAAACGTAAGCTTGCAACAGGTGATGAATTAACAACTGGTGTATTGAAAGTTGTTAAAGTTTACTTAGCTTCTTACCTGTTGAAGACATGCCACATGATGCTAACGGTGTGCCGGTAGATATCGTATTGAACCCGCTGGGTGTACCATCTCGTATGAACGTGGGTCAGATTCTAGAGACTCACTTGGGTATGGCGGCTAAAGGGCTTGGTGACAAAATCGAAAAAATGTTGAAAGAACAACGTACAGTTTTAGAACTGCGCGAATTCTTAGACAAGATTTATAACAAAGTCGGCGGTGAGCAAGAAGATCTTGATAGCTTGACTGATGAAGAAATTCTAGCGCTTGCAGGTAACTTGCGTGCGGGTGTGCCTTTAGCTACTCCTGTATTTGATGGTGCTGAAGAAAGTCAAATTAAAGACTTACTTGAATTGGCTGACATTTCACGTACTGGTCAAACAGTATTGTTTGACGGACGTACAGGTGAACAGTTTGACCGTCCAGTAACTGTTGG

>*Acinetobacter* 28207

AGCTCGTGAGCGTGTaATTCGTTTGCTTAAAGGCCAAGAGTCTAATGGCGGTGGTTCAACTAAACGTGGTGACAAACTCGTTGAAGAAGTGTTATCTGGTTTAGAGCTTGTTGATTTACTTGAAATTCAACCGGCAGATGAAGCAATCGCTGAGCGTTTAACTCAAATTCAAGTGTTCTTAAAAGAAAAGAGCGCAGAAATTGACGAGAAATTCGCTGAGAAGAAAGTAAGCTTGCAACAGGTGATGAATTAACAACTGGCGTATTGAAAGTTGTTAAAGTTTACTTAGCTGCCACACGATGCTAACGGTGTACCAGTAGATATCGTATTGAACCCGTTGGGCGTACCATCTCGTATGAACGTGGGTCAGATTCTTGAGACTCACTTAGGTATGGCGGCTAAAGGGCTTGGTGATAAAATCGAGAAAATGTTGAAAGAACAGCGTACAGTTTTAGAACTGCGTGAATTCTTAGACAAGATTTATAACAAAGTCGGTGGTGAGCAAGAAGATCTTGATAGCTTAACTGATGCTGAAGTCTTGGCACTTTCAGGCAACTTACGTGCAGGTGTACCTTTGGCTACTCCTGTATTCGATGGTGCTGAAGAAAGCCAAATTAAAGACTTGCTTGAGTTAGCTGGTATCTCTCGTACAGGTCAAACAGTATTGTTTGATGGCCGTACTGGTGAACAGTTTGATCGTCCTGTAACT

>*Acinetobacter* 30005

CTCGTGAGCGTGTAATTCGTTTGCTTAAAGGCCAAGAGTCTAATGGCGGTGGTTCAACTAAACGTGGTGACAAACTCGTTGAAGAAGTGTTATCTGGTTTAGAGCTTGTTGATTTACTTGAAATTCAACCGGCAGATGAAGCAATCGCTGAGCGTTTAACTCAAATTCAAGTGTTCTTAAAAGAAAAGAGCGCAGAAATTGACGAGAAATTCGCTGAGAAGAAACGTAAGCTTGCAACAGGTGATGAATTAACAACTGGCGTATTGAAAGTTGTTAAAGTTTACTTAGCTCTAACATCTTACCTGTTGAAGACATGCCACACGATGCTAACGGTGTACCAGTAGATATCGTATTGAACCCGTTGGGCGTACCATCTCGTATGAACGTGGGTCAGATTCTTGAGACTCACTTAGGTATGGCGGCTAAAGGGCTTGGTGATAAAATCGAGAAAATGTTGAAAGAACAGCGTACAGTTTTAGAACTGCGTGAATTCTTAGACAAGATTTATAACAAAGTCGGTGGTGAGCAAGAAGATCTTGATAGCTTAACTGATGCTGAAGTCTTGGCACTTTCAGGCAACTTACGTGCAGGTGTACCTTTGGCTACTCCTGTATTCGATGGTGCTGAAGAAAGCCAAATTAAAGACTTGCTTGAGTTAGCTGGTATCTCTCGTACAGGTCAAACAGTATTGTTTGATGGCCGTACTGGTGAACAGTTTGATCGTCCTGTAACTGTAGG

>*Acinetobacter* 31132

CTCGTGAGCGTGTAATTCGTTTGCTTAAAGGCCAAGAGTCTAATGGCGGTGGTTCAACTAAACGTGGTGACAAACTCGTTGAAGAAGTGTTATCTGGTTTAGAGCTTGTTGATTTACTTGAAATTCAACCGGCAGATGAAGCAATCGCTGAGCGTTTAACTCAAATTCAAGTGTTCTTAAAAGAAAAGAGCGCAGAAATTGACGAGAAATTCGCTGAGAAGAAACGTAAGCTTGCAACAGGTGATGAATTAACAACTGGCGTATTGAAAGTTGTTAAAGTTTACTTAGCTGTTAAACGTCGTATTCAGCCACACGATGCTAACGGTGTACCAGTAGATATCGTATTGAACCCGTTGGGCGTACCATCTCGTATGAACGTGGGTCAGATTCTTGAGACTCACTTAGGTATGGCGGCTAAAGGGCTTGGTGATAAAATCGAGAAAATGTTGAAAGAACAGCGTACAGTTTTAGAACTGCGTGAATTCTTAGACAAGATTTATAACAAAGTCGGTGGTGAGCAAGAAGATCTTGATAGCTTAACTGATGCTGAAGTCTTGGCACTTTCAGGCAACTTACGTGCTGGTGTACCTTTGGCTACTCCTGTATTCGATGGTGCTGAAGAAAGTCAAATTAAAGACTTGCTTGAGTTAGCTGGTATCTCTCGTACAGGTCAAACAGTATTGTTTGATGGCCGTACTGGTGAACAGTTTGATCGTCCTGTAACTGTAGGTTACATGTACA

>*Acinetobacter* 31205

CTCGTGAGCGTGTAATTCGTTTGCTTAAAGGCCAAGAGTCTAATGGCGGTGGTTCAACTAAACGTGGTGACAAACTCGTTGAAGAAGTGTTATCTGGTTTAGAGCTTGTTGATTTACTTGAAATTCAACCGGCAGATGAAGCAATCGCTGAGCGTTTAACTCAAATTCAAGTGTTCTTAAAAGAAAAGAGCGCAGAAATTGACGAGAAATTCGCTGAGAAGAAACGTAAGCTTGCAACAGGTGATGAATTAACAACTGGCGTATTGAAAGTTGTTAAAGTTTACTTAGCTGTTAAACGTCGTATTCATCTTACCTGTTGAAGACATGCCACACGATGCTAACGGTGTACCAGTAGATATCGTATTGAACCCGTTGGGCGTACCATCTCGTATGAACGTGGGTCAGATTCTTGAGACTCACTTAGGTATGGCGGCTAAAGGGCTTGGTGATAAAATCGAGAAAATGTTGAAAGAACAGCGTACAGTTTTAGAACTGCGTGAATTCTTAGACAAGATTTATAACAAAGTCGGTGGTGAGCAAGAAGATCTTGATAGCTTAACTGATGCTGAAGTCTTGGCACTTTCAGGCAACTTACGTGCAGGTGTACCTTTGGCTACTCCTGTATTCGATGGTGCTGAAGAAAGCCAAATTAAAGACTTGCTTGAGTTAGCTGGTATCTCTCGTACAGGTCAAACAGTATTGTTTGATGGCCGTACTGGTGAACAGTTTGATCGTCCTGTAACTGTAGG

>*Acinetobacter* 31357

GCTCGTGAGCGTGTAATTCGTTTGCTTAAAGGCCAAGAGTCTAATGGCGGTGGTTCAACTAAACGTGGTGACAAACTCGTTGAAGAAGTGTTATCTGGTTTAGAGCTTGTTGATTTACTTGAAATTCAACCGGCAGATGAAGCAATCGCTGAGCGTTTAACTCAAATTCAAGTGTTCTTAAAAGAAAAGAGCGCAGAAATTGATGAGAAATTCGCTGAGAAGAAACGTAAGCTTGCAACAGGTGATGAATTAACAACTGGCGTATTGAAAGTTGTTAAAGTTTACTTAGCTGTTAAACGTCGTATTCATCTTACCTGTTGAAGACATGCCACACGATGCTAACGGTGTACCAGTAGATATCGTATTGAACCCGTTGGGCGTACCATCTCGTATGAACGTGGGTCAGATTCTTGAGACTCACTTAGGTATGGCGGCTAAAGGGCTTGGTGATAAAATCGAGAAAATGTTGAAAGAACAGCGTACAGTTTTAGAACTGCGTGAATTCTTAGACAAGATTTATAACAAAGTCGGTGGTGAGCAAGAAGATCTTGATAGCTTAACTGATGCTGAAGTCTTGGCACTTTCAGGCAACTTACGTGCTGGTGTACCTTTGGCTACTCCTGTATTCGATGGTGCTGAAGAAAGCCAAATTAAAGACTTGCTTGAGTTAGCTGGTATCTCTCGTACAGGTCAAACAGTATTGTTTGATGGCCGTACTGGTGAACAGTTTGATCGTCCTGTAACTGTGGGTTAC

>*Acinetobacter* 31975

TCGTGAGCGTGTAATTCGTTTGCTTAAAGGCCAAGAGTCTAATGGCGGTGGTTCAACTAAACGTGGTGACAAACTCGTTGAAGAAGTGTTATCTGGTTTAGAGCTTGTTGATTTACTTGAAATTCAACCGGCAGATGAAGCAATCGCTGAGCGTTTAACTCAAATTCAAGTGTTCTTAAAAGAAAAGAGTGCAGAAATTGATGAGAAATTCGCTGAGAAGAAACGTAAGCTTGCAACAGGTGATGAACTAACAACTGGCGTATTGAAAGTTGTTAAAGTTTACTTAGCTGTCTTACCTGTTGAAGACATGCCACACGATGCTAACGGTGTACCGGTAGATATCGTATTGAACCCGTTGGGCGTACCATCTCGTATGAACGTGGGTCAGATTCTTGAGACTCACTTAGGTATGGCGGCTAAAGGGCTTGGTGATAAAATCGAGAAAATGTTGAAAGAACAGCGTACAGTTTTAGAACTGCGTGAATTCTTAGACAAGATTTATAACAAAGTCGGTGGTGAGCAAGAAGATCTTGATAGCTTAACTGATGCTGAAGTCTTGGCACTTTCAGGCAACTTACGTGCAGGTGTACCTTTAGCTACTCCTGTATTCGATGGTGCTGAAGAAAGCCAAATTAAAGACTTGCTTGAGTTAGCTGGTATCTCTCGTACAGGCCAAACAGTATTGTTTGATGGCCGTACTGGTGAACAGTTTGATCGTCCTGTAACTGTAGGTTACATGTACATG

>*Acinetobacter* 32493

CTCGTGAGCGTGTAATTCGTTTGCTTAAAGGCCAAGAGTCTAATGGCGGTGGTTCAACTAAACGTGGTGACAAACTCGTTGAAGAAGTGTTATCTGGTTTAGAGCTTGTTGATTTACTTGAAATTCAACCGGCAGATGAAGCAATCGCTGAGCGTTTAACTCAAATTCAAGTGTTCTTAAAAGAAAAGAGCGCAGAAATTGACGAGAAATTCGCTGAGAAGAAACGTAAGCTTGCAACAGGTGATGAATTAACAACTGGCGTATTGAAAGTTGTTAAAGTTTACTTAGCTGTTCTTACCTGTTGAAGACATGCCACACGATGCTAACGGTGTACCAGTAGATATCGTATTGAACCCGTTGGGCGTACCATCTCGTATGAACGTGGGTCAGATTCTTGAGACTCACTTAGGTATGGCGGCTAAAGGGCTTGGTGATAAAATCGAGAAAATGTTGAAAGAACAGCGTACAGTTTTAGAACTGCGTGAATTCTTAGACAAGATTTATAACAAAGTCGGTGGTGAGCAAGAAGATCTTGATAGCTTAACTGATGCTGAAGTCTTGGCACTTTCAGGCAACTTACGTGCTGGTGTACCTTTGGCTACTCCTGTATTCGATGGTGCTGAAGAAAGTCAAATTAAAGACTTGCTTGAGTTAGCTGGTATCTCTCGTACAGGTCAAACAGTATTGTTTGATGGCCGTACTGGTGAACAGTTTGATCGTCCTGTAACTGTAGGTTACA

>*Acinetobacter* 33007

GCTCGTGAGCGTGTAATTCGTTTGCTTAAAGGCCAAGAGTCTAATGGCGGTGGTTCAACTAAACGTGGTGACAAACTCGTTGAAGAAGTGTTATCTGGTTTAGAGCTTGTTGATTTACTTGAAATTCAACCGGCAGATGAAGCAATCGCTGAGCGTTTAACTCAAATTCAAGTGTTCTTAAAAGAAAAGAGCGCAGAAATTGACGAGAAATTCGCTGAGAAGAAACGTAAGCTTGCAACAGGTGATGAATTAACAACTGGCGTATTGAAAGTTGTTAAAGTTTACTTAGCTCTTACCTGTTGAAGACATGCCACATGATGCTAACGGTGTGCCGGTAGATATCGTATTGAACCCGCTGGGTGTACCATCTCGTATGAACGTGGGTCAGATTCTAGAGACTCACTTGGGTATGGCGGCTAAAGGGCTTGGTGACAAAATCGAAAAAATGTTGAAAGAACAACGTACAGTTTTAGAACTGCGCGAATTCTTAGACAAGATTTATAACAAAGTCGGTGGTGAGCAAGAAGATCTTGATAGCTTGACTGATGAAGAAATTCTAGCGCTTGCAGGTAACTTGCGTGCGGGTGTGCCTTTAGCTACTCCTGTATTTGATGGTGCTGAAGAAAGTCAAATTAAAGACTTACTTGAATTGGCTGACATTTCACGTACTGGTCAAACAGTATTGTTTGACGGACGTACAGGTGAACAGTTTGACCGTCCAGTAACTGT

>*Acinetobacter* 33291

CGGCTCGTGAGCGTGTAATTCGTTTGCTTAAAGGCCAAGAGTCTAATGGCGGTGGTTCAACTAAACGTGGTGATAAACTTTCTGAAGATTTATTATCTGGTTTAGAGCTTGTTGACTTACTTGAAATTCAACCAGCAGATGAAGCAATCGCTGAGCGTTTAACTCAAATTCAAGTGTTCTTAAAAGAGAAGAGCGCAGAAATCGATGAGAAATTCGCTGAGAAGAAACGTAAGCTTGCAACAGGTGATGAATTAACAACTGGCGTATTGAAAGTTGTTAAAGTTTACTTAGCTGTTAAACGTCGTATTCATCTTACCTGTTGAAGACATGCCACACGATGCTAACGGTGTACCAGTAGATATCGTATTGAACCCGTTGGGCGTACCATCTCGTATGAACGTGGGTCAGATTCTTGAGACTCACTTAGGTATGGCGGCTAAAGGGCTTGGTGATAAAATCGAGAAAATGTTGAAAGAACAGCGTACAGTTTTAGAACTGCGTGAATTCTTAGACAAGATTTATAACAAAGTCGGTGGTGAGCAAGAAGATCTTGATAGCTTAACTGATGCTGAAGTCTTGGCACTTTCAGGCAACTTACGTGCTGGTGTACCTTTGGCTACTCCTGTATTCGATGGTGCTGAAGAAAGTCAAATTAAAGACTTGCTTGAGTTAGCTGGTATCTCTCGTACAGGTCAAACAGTATTGTTTGATGGCCGTACTGGTGAACAGTTTGATCGTCCTGTAACTGTAGG

>*Acinetobacter* 33632

CTCGTGAGCGTGTAATTCGTTTGCTTAAAGGCCAAGAGTCTAATGGCGGTGGTTCAACTAAACGTGGTGACAAACTCGTTGAAGAAGTGTTATCTGGTTTAGAGCTTGTTGATTTACTTGAAATTCAACCGGCAGATGAAGCAATCGCTGAGCGTTTAACTCAAATTCAAGTGTTCTTAAAAGAAAAGAGCGCAGAAATTGATGAGAAATTCGCTGAGAAGAAACGTAAGCTTGCAACAGGTGATGAATTAACAACTGGCGTATTGAAAGTTGTTAAAGTTTACTTAGCTGTTATCTTACCTGTTGAAGACATGCCACACGATGCTAACGGTGTACCAGTAGATATCGTATTGAACCCGTTGGGCGTACCATCTCGTATGAACGTGGGTCAGATTCTTGAGACTCACTTAGGTATGGCGGCTAAAGGGCTTGGTGATAAAATCGAGAAAATGTTGAAAGAACAGCGTACAGTTTTAGAACTGCGTGAATTCTTAGACAAGATTTATAACAAAGTCGGTGGTGAGCAAGAAGATCTTGATAGCTTAACTGATGCTGAAGTCTTGGCACTTTCAGGCAACTTACGTGCTGGTGTACCTTTGGCTACTCCTGTATTCGATGGTGCTGAAGAAAGCCAAATTAAAGACTTGCTTGAGTTAGCTGGTATCTCTCGTACAGGTCAAACAGTATTGTTTGATGGCCGTACTGGTGAACAGTTTGATCGTCCTGTAACTGTGG

>*Acinetobacter* 33904

CGGCTCGTGAACGTGTAATTCGTTTACTTAAAGGTCAAGAGTCTAATGGCGGTGGTTCAACTAAACGTGGCGATAAACTTTCTGAAGATTTATTGTCTGGCTTAGAGCTTGTTGATTTACTTGAAATTCAACCGACAGATGAAGCAATCGCTGAGCGTTTAACTCAGATTCAAGTGTTCTTGAAAGAGAAGAGCGCAGAAATTGATGAGAAATTCGCTGAGAAGAAACGTAAGCTTGCAACAGGTGATGAGTTAACAACTGGCGTATTGAAAGTTGTTAAAGTTTACTTAGCTGTTAAACGTCGCCACACGATGCGAACGGTGTACCTGTAGATATCGTATTGAACCCGTTGGGCGTACCATCTCGTATGAACGTGGGTCAGATTCTCGAAACTCACTTGGGTATGGCGGCTAAAGGGCTTGGTGACAAAATCGAAAAAATGTTGAAAGAACAGCGTACAGTTTTAGAACTACGTGAATTCTTAGACAAGATTTATAACAAAGTCGGTGGCGAGCAAGAAGATCTTGATAGCTTGACTGATGATGAAATCCTAGCACTTTCAGGCAACTTGCGTGCTGGTGTTCCTTTGGCTACTCCTGTATTTGATGGTGCTGAAGAAAGTCAAATTAAAGATTTACTTGAGTTGGCTGACATTTCACGTACAGGTCAAACAGTATTGTTTGATGGACGTACAGGTGAACAGTTTGACCGTCCAGTAACTG

>*Acinetobacter* 34064

CGGCTCGTGAGCGTGTAATTCGTTTGCTTAAAGGCCAAGAGTCTAATGGCGGTGGTTCAACTAAACGTGGTGATAAACTTTCTGAAGATTTATTATCTGGTTTAGAGCTTGTTGACTTACTTGAAATTCAACCAGCAGATGAAGCGATCGCTGAGCGTTTAACTCAAATTCAAGTGTTCTTGAAAGAGAAGAGCGCAGAAATCGATGAGAAATTCGCTGAGAAGAAACGTAAGCTTGCAACAGGTGATGAATTAACAACTGGCGTATTAAAAGTTGTTAAAGTTTACTTAGCTGTTAAACGTCGTATTCACCACATGATGCTAACGGTGTGCCGGTAGATATCGTATTGAACCCGCTAGGTGTACCATCTCGTATGAACGTGGGTCAGATTCTAGAGACTCACTTGGGTATGGCGGCTAAAGGGCTTGGTGACAAAATCGAAAAAATGTTGAAAGAACAACGTACAGTTTTAGAACTGCGCGAATTCTTAGACAAGATTTATAACAAAGTCGGCGGTGAGCAAGAAGATCTTGATAGCTTGACTGATGAAGAAATTCTAGCGCTTGCAGGTAACTTGCGTGCGGGTGTGCCTTTAGCTACTCCTGTATTTGATGGTGCTGAAGAAAGTCAAATTAAAGACTTACTTGAATTGGCTGACATTTCACGTACGGGTCAAACAGTATTGTTTGACGGACGTACAGGTGAACAGTTTGACCGTCCAGTAACTGTTGGTTAC
